# Isolated Ocular Mpox without Skin Lesions, United States

**DOI:** 10.3201/eid2906.230032

**Published:** 2023-06

**Authors:** Minh T. Nguyen, Akshay Mentreddy, Julie Schallhorn, Matilda Chan, Su Aung, Sarah B. Doernberg, Jennifer Babik, Kevin Miles, Katherine Yang, Emily Lydon, Daniel J. Minter, John Gonzales, Jessica Shantha, Thuy Doan, Gerami D. Seitzman

**Affiliations:** University of California, San Francisco, California, USA (M.T. Nguyen, A. Mentreddy, J. Schallhorn, M. Chan, S. Aung, S.B. Doernberg, J. Babik, K. Miles, K. Yang, E. Lydon, D.J. Minter, J. Gonzales, J. Shantha, T. Doan, G.D. Seitzman);; Francis I. Proctor Foundation, San Francisco (M.T. Nguyen, A. Mentreddy, M. Chan, J. Gonzales, J. Shantha, T. Doan, G.D. Seitzman)

**Keywords:** mpox, viruses, monkeypox virus, orthoviruses, eye infection, California, United States

## Abstract

We report a case of a 53-year-old HIV-negative patient in San Francisco, California, USA, with no classic mpox prodromal symptoms or skin lesions who experienced fulminant, vision-threatening scleritis, keratitis, and uveitis. Deep sequence analysis identified monkeypox virus RNA in the aqueous humor. We confirmed the virus on the cornea and sclera by PCR.

We report a case of ocular-only mpox infection in a 53-year-old man in San Francisco, California, USA. His medical history included chronic lymphocytic leukemia (CLL), inactive 2 years after treatment with obinutuzumab and venetoclax but with persistent lymphopenia. He reported male sexual partners but was HIV negative. Symptoms in his right eye began August 2022 as itching and nasal scleral redness (Figure, panel A). There was no fever, rash, or lymphadenopathy. Eye redness worsened; the patient sought care at an urgent care facility and was given erythromycin ointment. Continued vision loss led to an emergency department visit, resulting in a diagnosis of preseptal cellulitis, treated with was trimethoprim/sulfamethoxazole plus amoxicillin/clavulanic acid.

**Figure:**
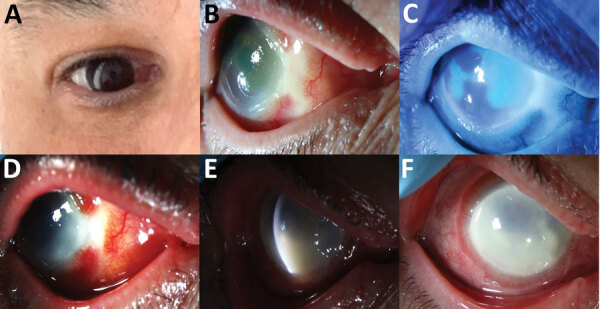
Clinical progression of ocular mpox in patient in California, USA. A) Initial manifestation of nasal scleral inflammation. B) Nasal scleral necrosis with surrounding scleritis. C) Corneal epithelial sloughing. D) Worsening scleritis and nasal keratitis. E) Corneal endothelial inflammatory plaque. Nasal area of corneal irregularity represents the area of biopsy. F) Progression of diffuse keratitis and corneal limbitis.

In early September 2022, the patient sought care at a county eye clinic for purulent conjunctivitis and corneal epithelial defects. Clinicians suspected gonococcal conjunctivitis and administered intramuscular ceftriaxone and topical moxifloxacin 0.5%. Bacterial and fungal ocular cultures and herpetic viral PCR returned negative results. Topical prednisolone acetate 1% and oral valacyclovir failed to control the eye inflammation. Three weeks after initial symptoms appeared, the patient’s ocular inflammation increased; keratic precipitates and a moderate corneal opacity developed. Uveitis and scleritis workups did not yield a specific diagnosis (Table).

**Table:** Summary of laboratory and microbiology testing of patient with ocular mpox, California, USA

Test	Result
Laboratory and microbiology testing from serum or ocular surface scraping or swab specimen	
Human leukocyte antigen B27	Negative
Angiotensin-converting enzyme	Within reference range
Complement	Within reference range
Antineutrophilic cytoplasmic antibody	Within reference range
Antinuclear antibodies	Within reference range
Rheumatoid factor	Within reference range
Anti-cyclic citrullinated peptide	Within reference range
HIV antibody/antigen	Negative ×2
QuantiFERON-TB Gold*	Negative
Fluorescent treponemal antibody absorption	Negative ×2
Herpes simplex virus 1 DNA from ocular surface swab specimen	Negative ×2
Cytomegalovirus DNA from ocular surface swab specimen	Negative ×2
Varicella zoster virus DNA from ocular surface swab specimen	Negative ×2
Bacterial culture on blood, chocolate, Middlebrook agar	Negative ×2
Fungal culture on potato flake agar	Negative ×2
Monkeypox virus RNA from aqueous sample	Positive for MPXV
Monkeypox virus DNA from conjunctival swab specimen	Positive ×5
Testing from scleral and corneal biopsy	
Periodic acid Schiff for fungus	Negative for fungal organisms
Grocott’s methamine silver	Negative for fungal organisms
Steiner	Negative for spirochetes
Acid-fast bacteria (Fite)†	Negative for acid-fast organisms
* Trepona pallidum *antibody	Negative
Herpes simplex virus 1 & 2	Negative
Varicella zoster virus	Negative
Complete blood count with differential obtained at the time of hospitalization	
Red blood cell count (reference 4.4–5.9 ×10^12^ cells/L)	4.45 × 10^12^ cells/L
Hemoglobin (reference 13.6–17.5 g/dL)‡	13.7 g/dL
Platelets (reference140–450 ×10^9^/L)‡	174 × 10^9^/L
Leukocyte count (reference 3.4–10 ×10^9^ cells/L)	4.3 × 10^9^ cells/L
Absolute neutrophils (reference 1.8 × 6.8 × 10^9^ cells/L)	3.93 × 10^9^ cells/L
Absolute monocytes (reference 0.2–0.8 ×10^9^ cells/L)‡§	0.06 × 10^9^ cells/L
Absolute eosinophils (reference 0–0.4 ×10^9^ cells/L)	0
Absolute basophils (reference 0–0.1 × 10^9^ cells/L)	0.01 × 10^9^ cells/L
Absolute lymphocytes (reference 1–3.4 × 10^9^ cells/L)‡§	0.33 × 10^9^ cells/L

*QIAGEN, https://www.qiagen.com. †Newcomer Supply, https://www.newcomersupply.com/product/afb-fite-stain-kit. ‡Hemoglobin (>10 g/dL) and platelet count (>100 × 10^9^/L) do not meet criteria for active chronic lymphocytic leukemia and treatment. §Indicates chronic lymphocytopenia.

In late September 2022, at a second opinion examination at a University of California clinic, the patient’s right eye acuity was 20/640. Examination showed a nasal patch of avascular scleral necrosis (Figure, panel B), and corneal epithelial sloughing (Figure, panel C) with microcystic edema. Repeat ocular surface cultures and PCR were negative. Given the negative results of extensive infectious etiology testing, we prescribed oral prednisone (40 mg/d) for presumed undifferentiated necrotizing anterior scleritis and keratitis.

One week later, corneal inflammation worsened (Figure, panel D). Clinical deterioration on systemic steroids continued to raise suspicion for ocular infection; we stopped steroid treatments. Again, cultures and PCR remained negative. Without prednisone, the patient’s limbal infiltrates worsened, with progressive corneal haze. White corneal endothelial plaques appeared. We performed anterior chamber paracentesis for cultures, viral PCR, and an RNA deep-sequencing (RNA-seq) protocol previously described (*1*). The patient’s scleritis, keratitis, and anterior uveitis worsened (Figure, panels E, F). Right eye vision decreased to hand motion only. We performed a diagnostic scleral and corneal biopsy and initiated voriconazole for presumed fungal infection. The biopsy results returned negative for infection, and voriconazole was stopped.

Given the progressive clinical disease and negative infectious workup, we processed residual aqueous fluid for RNA-seq, which revealed a high number of reads aligning to monkeypox virus (MPXV) (Appendix Figure 1). Orthogonal testing from corneal and scleral swab specimens also returned positive results for MPXV by PCR. A nasal swab specimen obtained on the same day tested negative for MPXV by PCR.

When presented with those findings (Table), the patient offered additional information regarding high-risk sexual activities, previously withheld because his definition of sexual activity included only penetrative intercourse. He described an encounter with a male partner with semen deposition into his right eye 2 weeks before the onset of his symptoms. He received the first dose of mpox vaccine for higher risk groups the week after this encounter. Several months later, during casual text messaging, the partner from this activity revealed a subsequent penile mpox diagnosis. (Appendix Figure 2). 

Following treatment guidance from the Centers for Disease Control and Prevention, we initiated oral tecovirimat (600 mg 2×/d), topical trifluridine (6×/d), and weekly topical ophthalmic betadine washes (*2*). Four months into this course, worsening vision to light perception and repeat positive conjunctival mpox PCR led to inpatient admission for intravenous tecovirimat and cidofovir. Used in a compassionate-use capacity, tecovirimat and cidofovir have shown good activity against other orthopoxviruses, and in vitro and animal models have shown the antivirals to be effective against mpox (*3*). Unfortunately, after 3 weeks of inpatient treatment, the ocular disease did not improve. The patient’s eye remained PCR positive for MPXV with an opacified and vascularized cornea.

Ocular manifestations of mpox during the 2010–2013 outbreak were associated with a more severe systemic presentation (*4*). However, during the recent 2022 outbreak, ocular findings do not appear to have correlated with systemic disease severity (*5*–*7*). Isolated ocular mpox in the absence of systemic or skin findings is exceedingly rare.

In conclusion, we describe a case of isolated ocular mpox with no skin lesions or systemic prodromal symptoms in a relatively immunocompromised patient. Because pertinent history and clinical suspicion were lacking, metagenomic RNA sequencing was highly valuable in helping identify the pathogen. Semen was the likely vehicle for direct transmission (*8*), although hand-to-eye contact with unobserved lesions or exposure through nondisclosed encounters cannot be excluded. Intraocular mpox involving the sclera, cornea, and anterior chamber, along with persistently PCR-positive ocular surface mpox, has a poor visual prognosis. There is no established treatment for ocular mpox (*9*). Continued mpox disease progression over several months is unusual and could raise suspicion of immunosuppression or treatment resistance.

Appendix Additional information about a case of ocular mpox, California, USA.
