# Supplementary material for: Isolated Ocular Mpox without Skin Lesions, United States
Source: Emerg Infect Dis. 2023 Jun;29(6):1285–8. doi: 10.3201/eid2906.230032 (PMC10202873; doi:10.3201/eid2906.230032)
Supplement: Appendix — Additional information about a case of ocular mpox, California, USA. [file 23-0032-Techapp-s1.pdf]

Article DOI: https://doi.org/10.3201/eid2906.230032

Isolated Ocular Mpox without Skin Lesions, United States

Appendix

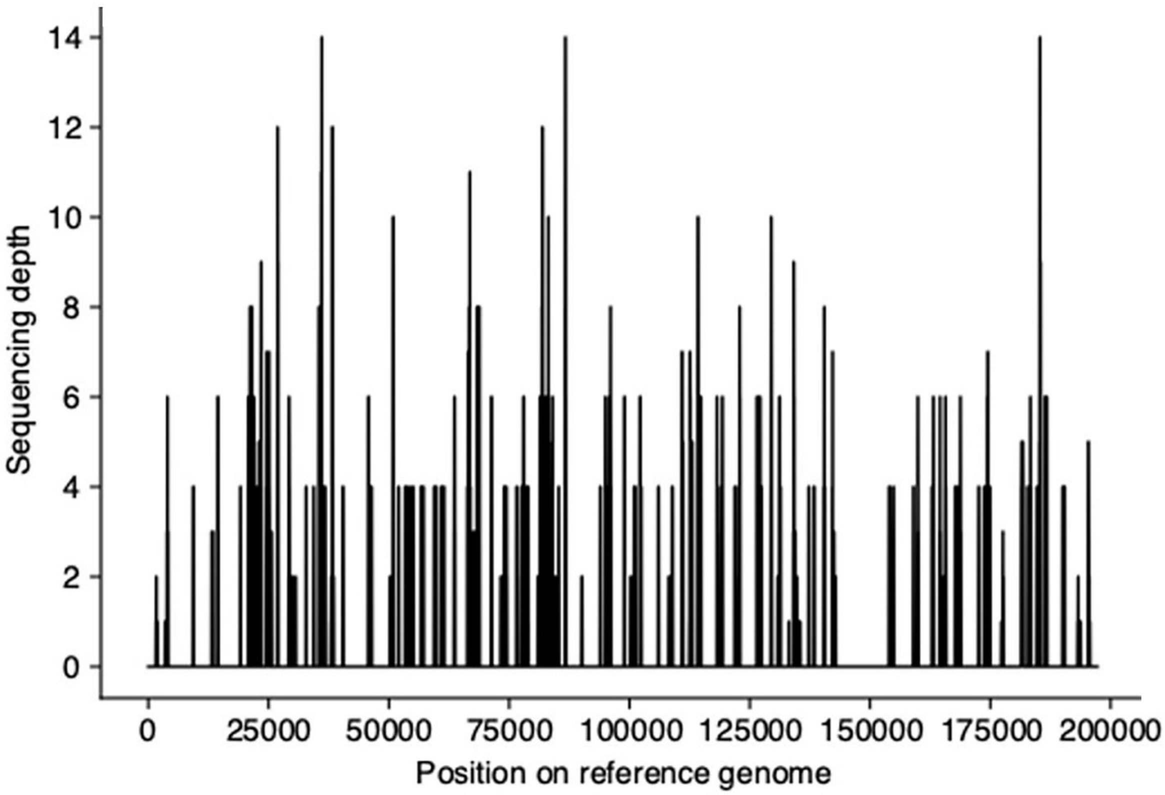

Appendix Figure 1. Identification of monkeypox virus in the patient's aqueous fluid by metagenomic RNA deep sequencing (accession no. PRJNA9554436). Alignment of detected MPXV reads to the MPXV genome (NCBI reference sequence ON563414.3). MPXV, monkeypox virus.

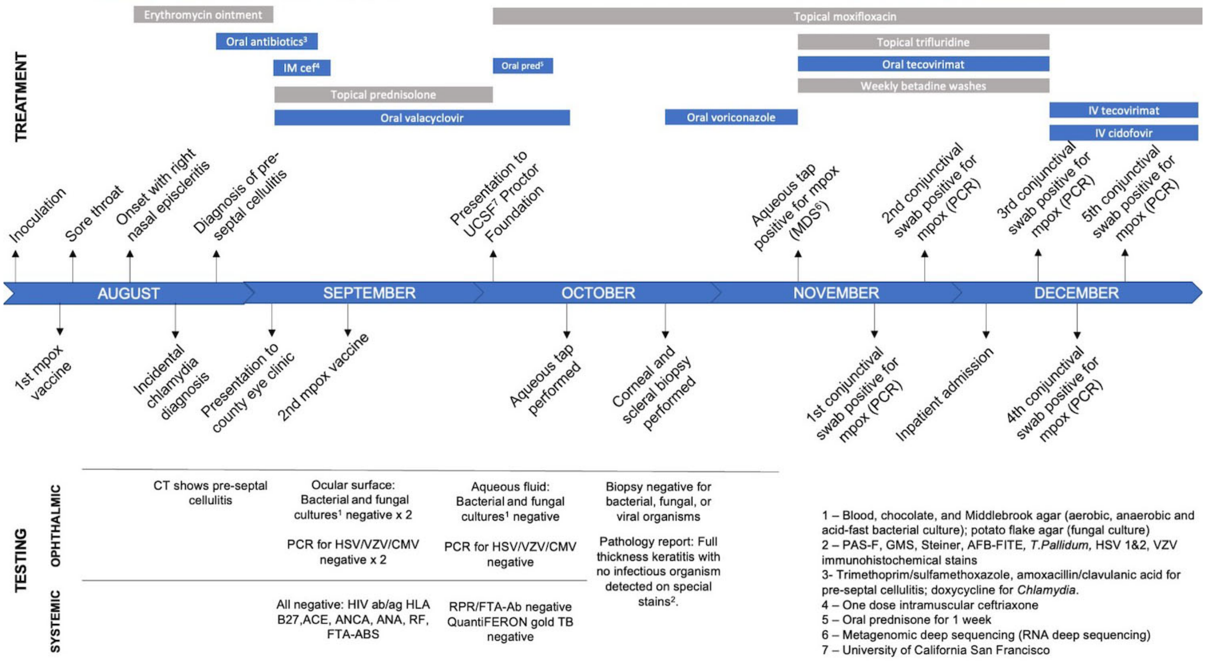

Appendix Figure 2. Clinical timeline of symptom onset, disease progression, important laboratory investigation and treatment.
